# Construction of tissue-engineered bone using a bioreactor and platelet-rich plasma

**DOI:** 10.3892/etm.2014.1774

**Published:** 2014-06-11

**Authors:** DONG WANG, HONGLEI JIANG, SHUZHEN WANG, HUIBO LI, HUAWU ZHANG, LEI ZHAO, TAO PENG, ZHONG CAO, SHUI SUN

**Affiliations:** 1 Department of Orthopaedics, Provincial Hospital Affiliated to Shandong University, Jinan, Shandong 250022, P.R. China; 2 Department of Cardiology, Provincial Hospital Affiliated to Shandong University, Jinan, Shandong 250022, P.R. China; 3 Department of Equipment, The People’s Hospital of Laiwu, Laiwu, Shandong 271100, P.R. China

**Keywords:** platelet-rich plasma, β-tricalcium phosphate, rabbit bone marrow mesenchymal stem cells, perfusion bioreactor

## Abstract

The aim of the present study was to construct tissue-engineered bone using a bioreactor and platelet-rich plasma (PRP). Bone marrow mesenchymal stem cells (BMSCs) and β-tricalcium phosphate (β-TCP) were cultured in a perfusion bioreactor with PRP-containing medium for 21 days to form a BMSC-TCP composite. Rabbits were then implanted with the BMSC-TCP composite. The morphology of the implanted BMSC-TCP composite was observed three months after surgery by scanning electron microscopy and hematoxylin and eosin (H&E) staining. In addition, the expression of cluster of differentiation (CD)31 and von Willebrand factor (WF) in the implanted BMSC-TCP composite was detected using immunohistochemistry. Bone formation was determined by comprehensive testing Following culture in a perfusion bioreactor and PRP, the BMSCs adhered to the β-TCP scaffold and the secretion of extracellular matrix was observed. The spreading and proliferation of cells was found to be enhanced on the scaffold. Furthermore, the vascular endothelial cell markers CD31 and VEF, were positively expressed. Therefore, these results suggest that tissue-engineered bone may be constructed using a bioreactor and PRP. PRP, which contains multiple growth factors, may promote vascularization of tissue-engineered bone.

## Introduction

Avascular necrosis of the femoral head is usually caused by a lack of osteoblast progenitor cells and blood vessels (1). The formation of new blood vessels in the bone necrosis region is a prerequisite for bone tissue repair. Growth factors have an important role in the construction of bone by tissue engineering. Previous studies have focused on the role of single growth factors in bone tissue engineering (2,3). However, the synergistic effects of various growth factors have rarely been studied. Furthermore, growth factors are usually prepared by genetic recombination, which has certain shortcomings, including difficulty of preparation, high cost and immunogenicity.

In the present study, platelet-rich plasma (PRP) was used as a source of growth factors. PRP is an autologous platelet concentrate and is obtained from fresh whole blood by centrifugation. The biological activity of PRP depends on the enrichment in platelets. It has been confirmed that PRP prepared by appropriate technology contains a variety of growth factors at high concentrations, including platelet-derived growth factor, transforming growth factor-β, vascular endothelial growth factor, insulin-like growth factor and epidermal growth factor. PRP has an important role in promoting the proliferation of bone precursor cells in various animals, including rabbits and goats (4,5). In addition, it promotes the proliferation of bone marrow mesenchymal stem cells (BMSCs) and their differentiation into osteoblasts (6). The growth factors contained in PRP have many functions, including promoting angiogenesis and accelerating the formation of collagen, thus promoting tissue healing and bone formation. The composition of growth factors released by PRP is consistent with that of human growth factors. Therefore, PRP is considered as an ideal source of growth factors for clinical application. PRP is primarily used in the repair of bone defects in the oral cavity and maxillofacial region; however, few studies have investigated the use of PRP in bone tissue engineering.

Marx *et al* (7) first used PRP in combination with autologous bone to promote new bone formation. Kim *et al* (8) combined PRP with β-tricalcium phosphate (β-TCP) and implanted the composite into skull defects in athymic mice, which resulted in a significant improvement in bone formation. The authors hypothesized that the application of inorganic materials as scaffolds requires the growth factors released by PRP, which may promote vascularization. In a study by Yokota *et al* (9) a model of rabbit iliac bone necrosis was established using liquid nitrogen, and a single injection of PRP was observed to accelerate the vascularization of necrotic bone. Tao *et al* (10) utilized a composite of TCP and PRP to repair ischemic necrosis of the femoral head in a rabbit model, and demonstrated that TCP-PRP composite materials not only provide a scaffold for osteoblasts, but also promote necrotic bone repair. Latalski *et al* (11) retrospectively studied 19 patients undergoing limb lengthening with an external fixator. The study showed that the treatment time for patients who received PRP was significantly shorter compared with that of the control group, and that the treatment had satisfactory clinical results. In addition, Curi *et al* (12) treated 25 cases of bisphosphonate-related osteonecrosis of the jaw (BRONJ) by resecting the necrotic bone and placing autologous PRP in the bone resection region. It was found that treatment with PRP was an effective strategy for the treatment of the patients with BRONJ. Furthermore, Scala *et al* (13) reported that the injection of PRP may be used as an effective method for repairing osteoradionecrosis.

In the present study, tissue-engineered bone was constructed by co-culturing BMSCs and β-TCP in a three-dimensional perfusion bioreactor. The BMSC-TCP composite was then implanted into rabbits and vascularization of the composite was evaluated *in vivo*.

## Materials and methods

### Reagents and instruments

Rabbit BMSCs were purchased from Cyagen Biosciences Inc. (Santa Clara, CA, USA). β-TCP was obtained from Shanghai Bio-lu Biomaterials Co., Ltd. (Shanghai, China). Hematoxylin and eosin (H&E), anti-cluster of differentiation (CD)31 antibody, anti-von Willebrand factor (vWF) antibody and 3,3′-diaminobenzidine (DAB) chromogenic reagent for immunohistochemistry were purchased from Boster Biological Technology Co., Ltd. (Wuhan, China). Low-glucose Dulbecco’s modified Eagle’s medium (DMEM) was obtained from HyClone (Rockford, IL, USA).

The perfusion bioreactor used in this study was jointly designed by our group and the East China University of Science and Technology (Shanghai, China). The whole system consisted of a MasterFlex peristaltic pump (Cole-Parmer Company, Vernon Hills, IL, USA), kcal head, perfusion column (including seals and sample slots), air filters, two silicone tubes, a three-way connector and cell culture flasks. Two silicone tubes were placed into the cell culture flask. One silicone tube was immersed in the culture medium in order to extract the culture medium. The tube was then connected to the perfusion column to transport the medium. The culture medium flowed back into the flask through another silicon tube. The JSM-T300 scanning microscope was obtained from JEOL-Technics Co. (Tokyo, Japan) and the light microscope was from Olympus Optical Co., Ltd. (Tokyo, Japan).

### Animals

Ten male New Zealand rabbits, 2 months old, with a mean weight of 3.50±0.30 kg, were obtained from the Experimental Animal Center of Shandong Province (Jinan, China). They were kept under standard conditions with free access to food and water.

All animal experiments were approved by Animal Care and Use Committee of Shandong University and were conducted according to the ethical guidelines of Shandong University (Jinan, China).

### Preparation of PRP gel

The rabbits were anesthetized and 5 ml blood was collected from the ear vein. The blood was mixed with 1 ml sodium citrate anticoagulant. Then, the mixture was centrifuged at 549 × g for 6 min. Following centrifugation, the mixture was separated into a supernatant and a red blood cell layer, with an interface between them. The supernatant and the red blood cells 1–2 mm below the interface were transferred to another centrifuge tube and were centrifuged again (454 × g, 6 min). The supernatant was discarded and the remaining solution (~0.8 ml) was PRP. A total of 0.8 ml PRP was then mixed with 0.2 ml anticoagulant (consisting of 1 ml 10% calcium chloride and l,000 units thrombin) and incubated for 10 sec in order to form a gel. The prepared PRP gel was then added to 25 ml culture medium to prepare PRP gel culture medium.

### Construction of BMSC-TCP composite using a bioreactor

Rabbit BMSCs were cultured in low-glucose DMEM to the third generation and then the cells were adjusted to a single cell suspension of 5xl0^6^ cells/ml. The β-TCP scaffold was immersed in the cell suspension and the scaffold was then turned upside down to allow the cells to enter into the pores of the scaffold. The β-TCP scaffold was then cultured at 37°C in an incubator at 5% CO_2_. After 1 day, the BMSC-TCP composite (β-TCP scaffold covered with cells) was placed into the perfusion bioreactor column, with all the interfaces sealed by silicone. The entire system was then placed into an incubator at 37°C containing 5% CO_2_ and cultured for 21 days. The flow rate was maintained at 3 ml/min. By measuring the content of glucose in the culture medium, the culture medium was replaced every 2–3 days. The BMSC-TCP composite was examined for pathological morphology and used for further animal experiments.

### Experimental grouping

Each rabbit was implanted with β-TCP and four different combinations of BMSCs and β-TCP, each at a different site. The five implanted materials were as follows: group A, β-TCP only; group B, uncultured BMSCs and β-TCP; group C, bioreactor-cultured BMSCs and β-TCP; group D, PRP-cultured BMSCs and β-TCP; and group E, bioreactor- and PRP-cultured BMSCs and β-TCP.

### BMSC-TCP composite implantation

The New Zealand white rabbits were anesthetized and placed in a left lateral position to fix their head and limbs. The right side was longitudinally incised to expose the superficial fascia. The BMSC-TCP composite was implanted between the subcutaneous superficial fascia and deep fascia. The position was recorded and the skin was sutured. The New Zealand rabbits were then kept under standard conditions and intramuscularly injected with 800,000 units penicillin each day, for 3 days following the surgery. The daily diet and wound healing were observed following the surgery.

### H&E staining

Three months following surgery, the BMSC-TCP composite was removed, and rinsed with saline. Then, the BMSC-TCP composite was fixed in 10% formalin, embedded in paraffin and cut into tissue sections. The tissue sections were dewaxed in xylene and rehydrated in graded alcohols. Following washing with running water and distilled water, the sections were stained with hematoxylin. After washing again, the sections were differentiated. Then, the sections were stained with eosin after washing with running water. Following dehydration and differentiation in alcohol, the sections were mounted and observed under a JSM-T300 scanning electron microscope.

### Immunohistochemistry

The expression levels of CD31 and vWF in the BMSC-TCP composites were detected using immunohistochemistry. Briefly, samples were fixed in formaldehyde and embedded in paraffin. Sections were dewaxed, rehydrated in graded alcohols and processed, prior to incubation with antibodies. After blocking, the sections were incubated with primary antibodies against CD13 and vWF. The sections were washed with phosphate-buffered saline, secondary antibodies (Santa Cruz Biotechnology, Inc., Santa Cruz, CA, USA) were then added and the sections were further incubated. The sections were then developed using the DAB chromogenic reagent and sections were counterstained with hematoxylin. Positive staining was presented as brown granules in cells. Five fields were randomly selected at high magnification under the light microscope and the number of positively stained cells was counted. The percentage of positive cells was the ratio of the number of positive cells to the number of total cells (≥26 cells were counted).

The scoring criteria were as follows. Scores based on the percentage of positive cells: 0, percentage of positive cells ≤25%; 1, percentage of positive cells between 25 and 50%; 2, percentage of positive cells between 50 and 75%; and 3, percentage of positive cells ≥75%. Scores based on intensity of staining: 0, no positive staining; 1, weak (pale yellow) staining; 2, medium (brown) staining; and 3, strong (tan) staining. The histological score was the sum of the above two types of score, and a histological score of 0 was expressed as negative, a score 2 or 3 as weak positive, a score 4 or 5 as medium positive and score 6 or 7 as strongly positive.

### Statistical analysis

The SPSS statistical software package, version 17.0 was used for statistical analyses (SPSS, Inc., Chicago, IL, USA). Data are presented as the mean ± standard deviation. Analysis of variance was performed to compare differences between the different groups. P<0.05 was considered to indicate a statistically significant difference.

## Results

### Morphological analysis of BMSC-TCP composite

**Figure 1:**
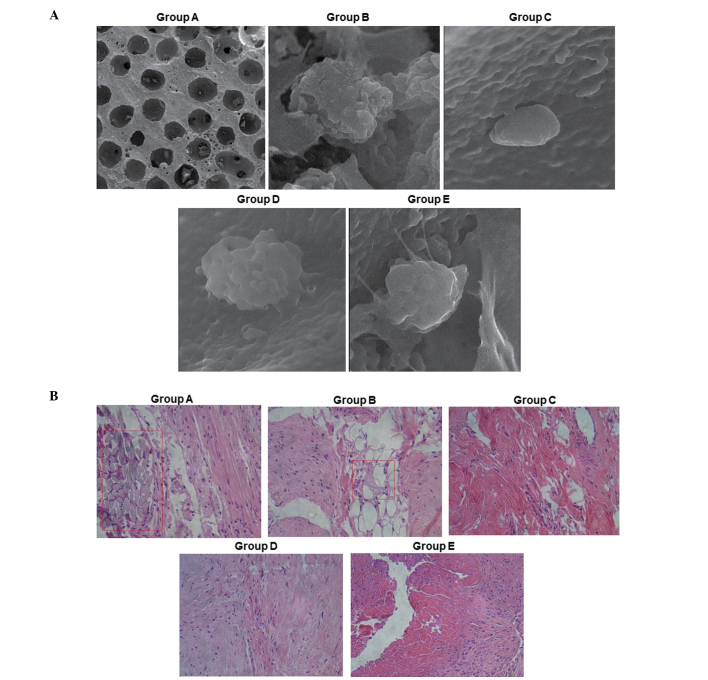
Morphological analysis of β-TCP scaffold and BMSC-TCP composite. Each rabbit was implanted at 5 different sites with β-TCP only (group A), BMSCs and β-TCP (group B), bioreactor-cultured BMSCs and β-TCP (group C), PRP-cultured BMSCs and β-TCP (group D), and bioreactor- and PRP-cultured BMSCs and β-TCP (group E), respectively. Three months following implantation, samples were removed from the rabbits (n=10). The morphology was observed using a JSM-T300 scanning electron microscope following H&E staining. (A) Representative scanning electron microscopy results for groups A–E. Magnification, 75kv × 5k. (B) Representative H&E staining results for groups A–E. Magnification, ×25. TCP, tricalcium phosphate; BMSCs, bone marrow mesenchymal stem cells; PRP, platelet-rich plasma; H&E, hematoxylin and eosin.

Composite materials were implanted between the superficial fascia and deep fascia under the skin. For implantation, each rabbit was implanted at 5 different sites with β-TCP only (group A), BMSCs and β-TCP (group B), bioreactor-cultured BMSCs and β-TCP (group C), PRP-cultured BMSCs and β-TCP (group D), and bioreactor- and PRP-cultured BMSCs and β-TCP (group E), respectively. Following implantation, the wounds all healed, without any swelling, effusion and signs of infection. Three months following implantation, the BMSC-TCP composites were removed. To observe the morphology of the BMSC-TCP composites-, scanning electron microscopy and H&E staining was performed. Representative scanning electron microscopy results are shown in Fig. 1A. The β-TCP blank scaffold showed an irregular porous structure, with an average pore size of 500–600 μm, a porosity of 75±10% and a stomatal communication rate of 100%. Observation of the BMSC-TCP composite under the scanning electron microscope revealed that BMSCs adhered on the β-TCP ceramic scaffold and the secretion of extracellular matrix was also observed. The spreading and proliferation of cells was good on the scaffold, indicating that the affinity of BMSCs to the β-TCP scaffold was high. Statistically, the number of adherent BMSCs in group E was significantly higher compared with that in the other groups (data not shown).

Representative H&E staining results for the various groups are shown in Fig. 1B. The results of the H&E staining demonstrate that there was more hyperblastosis in group E than in the other groups. Additionally, dense connective tissues were identified in group E, which are suggestive of bone formation. The βTCP scaffold and the tissue were tightly bound and vascular-like tissue was observed. However, in groups B, C, D and E, vascular-like tissue was rarely observed.

### Vascularization analysis by immunohistochemistry

**Figure 2:**
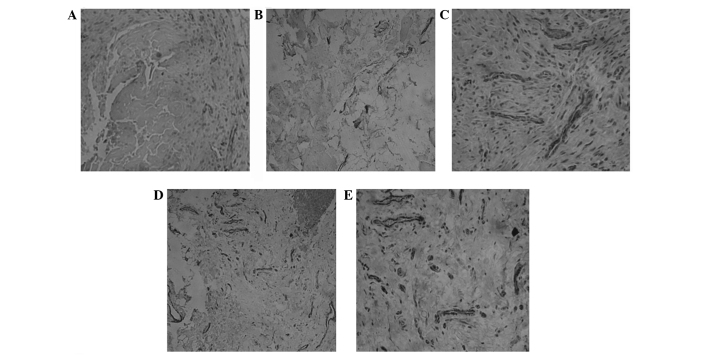
Immunohistochemical analysis of CD31 expression. Each rabbit was implanted at five different sites with β-TCP only (group A), BMSCs and β-TCP (group B), bioreactor-cultured BMSCs and β-TCP (group C), PRP-cultured BMSCs and β-TCP (group D), and bioreactor- and PRP-cultured BMSCs and β-TCP (group E), respectively. Three months following implantation, samples were collected from implanted rabbits (n=10). Expression of CD31 was analyzed using immunohistochemistry. Cells with brown granules were considered positive cells. Representative immunohistochemical results from (A) group A, (B) group B, (C) group C, (D) group D and (E) group E (magnification, ×200). CD, cluster of differentiation; TCP, tricalcium phosphate; BMSCs, bone marrow mesenchymal stem cells; PRP, platelet-rich plasma.

**Figure 3:**
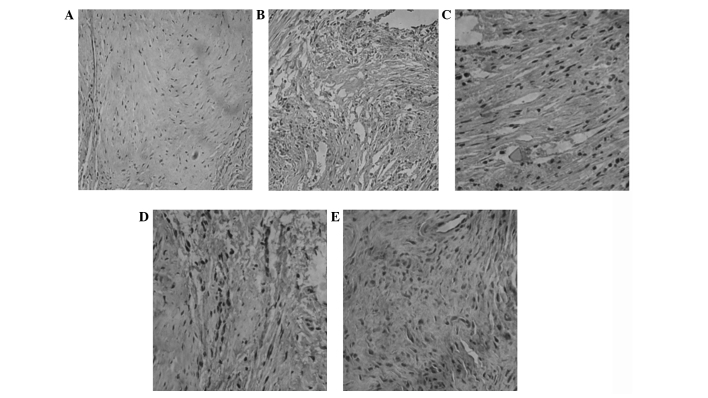
Immunohistochemical analysis of vWF expression. Each rabbit was implanted at five different sites with β-TCP only (group A), BMSCs and β-TCP (group B), bioreactor-cultured BMSCs and β-TCP (group C), PRP-cultured BMSCs and β-TCP (group D), and bioreactor- and PRP-cultured BMSCs and β-TCP (group E), respectively. Three months following implantation, samples were collected from implanted rabbits (n=10). vWF expression was detected using immunohistochemistry. Cells with brown granules were considered positive cells. Expression of vWF was analyzed by immunohistochemistry. Representative immunohistochemical results from (A) group A, (B) group B, (C) group C, (D) group D and (E) group E (magnification, ×200). vWF, von Willebrand Factor; TCP, tricalcium phosphate; BMSCs, bone marrow mesenchymal stem cells; PRP, platelet-rich plasma.

CD31 and vWF are expressed in vascular endothelial cells. Therefore, to evaluate the vascularization in the BMSC-TCP composite, immunohistochemical staining of CD31 and vWF was performed. Representative immunohistochemical staining results for CD31 and vWF are shown in Figs. 2 and 3, respectively. Cells with brown granules were positively stained cells. As shown in Figs. 2A and 3A, no positively stained cells were visible in group A. In groups B (Figs. 2B and 3B), C (Figs. 2C and 3C) and D (Figs. 2D and 3D), there were smaller brown particles observed within the cells, and these brown particles were unevenly distributed. However, in group E (Figs. 2E and 3E), a large number of brown-stained granules were observed under the microscope, with larger particles also observed. Histological scoring was conducted as described in Materials and methods and the scores for CD31 in each group are presented in Table I.

## Discussion

In the present study, BMSCs were three-dimensionally cultured in a perfusion bioreactor. This culture model has certain advantages compared with traditional planar two-dimensional culture models. Firstly, the culture medium in traditional two-dimensional cell culture models requires intermittent replacement, whilst in a three-dimensional bioreactor the culture medium flows, and therefore does not need to be replaced frequently. However, the flow medium may stimulate cells mechanically and simulate an *in vivo* cell stress environment. Therefore, the mechanical characteristics of the cells are similar to the biological changes that occur *in vivo* (14–16). In addition, cells may be fully combined with or adhered to the three-dimensional scaffold materials in the bioreactor, which means that the cell culture is similar to a three-dimensional growth (17,18). Furthermore, vascularized bone constructed by a three-dimensional culture model has better physiological functions and mechanical properties compared with bone constructed using a two-dimensional culture model.

In our previous study (19), BMSCs were used as seed cells and β-TCP as a scaffold material, and a tissue-engineered osteochondral composite was successfully constructed in a perfusion bioreactor. This composite was used in the repair of osteochondral defects in beagles. It was found that certain grafts did not completely integrate into the areas of bone cartilage defect, indicating that the constructed osteochondral composite was not completely vascularized. In the present study, in the process of bone construction in the three-dimensional perfusion bioreactor, autologous PRP was used as a cytokine source to promote bone formation and vascularization. The results demonstrated that the number of blood vessels in the composite cultured using the three-dimensional bioreactor and PRP was significantly higher compared with that in the other control groups. Furthermore, autologous PRP is a convenient, economical and sustainable source of high-quality cytokines.

## Figures and Tables

**Table I:** Results of histological scoring in each group for CD31.

Case number	Group A	Group B	Group C	Group D	Group E
1	0	2	3	4	6
2	1	2	3	3	6
3	0	3	2	5	7
4	2	4	4	6	7
5	1	1	3	3	6
6	3	2	5	5	5
7	2	3	2	5	4
8	1	4	1	6	6
9	3	2	3	7	4
10	3	3	2	5	5

Group A (n=10), β-TCP only; group B (n=10), BMSCs and β-TCP; group C (n=10), bioreactor-cultured BMSCs and β-TCP; group D (n=10), PRP-cultured BMSCs and β-TCP; and group E (n=10), bioreactor- and PRP-cultured BMSCs and β-TCP. CD, cluster of differntiation; TCP, tricalcium phosphate; BMSCs, bone marrow mesenchymal stem cells; PRP, platelet-rich plasma.

## References

[1] Song HJ, Lan BS, Cheng B (2011). Treatment of early avascular necrosis of femoral head by small intestinal submucosal matrix with peripheral blood stem cells. Transplant Proc.

[2] Feng L, Wu H, EL (2013). Effects of vascular endothelial growth factor 165 on bone tissue engineering. PLoS One.

[3] Zou D, Zhang Z, Ye D (2011). Repair of critical-sized rat calvarial defects using genetically engineered bone marrow-derived mesenchymal stem cells overexpressing hypoxia-inducible factor-1α. Stem Cells.

[4] Marques LF, Stessuk T, Camargo IC, Sabeh N, Santos LD, Ribeiro-Paes JT (2014). Platelet-rich plasma (PRP): Methodological aspects and clinical applications. Platelets.

[5] Kim TH, Kim SH, Sándor GK, Kim YD (2014). Comparison of platelet-rich plasma (PRP), platelet-rich fibrin (PRF), and concentrated growth factor (CGF) in rabbit-skull defect healing. Arch Oral Biol.

[6] Lin BN, Whu SW, Chen CH (2013). Bone marrow mesenchymal stem cells, platelet-rich plasma and nanohydroxyapatite-type I collagen beads were integral parts of biomimetic bone substitutes for bone regeneration. J Tissue Eng Regen Med.

[7] Marx RE, Carlson ER, Eichstaedt RM, Schimmele SR, Strauss JE, Georgeff KR (1998). Platelet-rich plasma: Growth factor enhancement for bone grafts. Oral Surg Oral Med Oral Pathol Oral Radiol Endod.

[8] Kim ES, Kim JJ, Park EJ (2010). Angiogenic factor-enriched platelet-rich plasma enhances in vivo bone formation around alloplastic graft material. J Adv Prosthodont.

[9] Yokota K, Ishida O, Sunagawa T (2008). Platelet-rich plasma accelerated surgical angio-genesis in vascular-implanted necrotic bone: an experimental study in rabbits. Acta Orthop.

[10] Tao H, Zhang C, Zeng B, Yuan T, Xu J, Song W (2005). Experimental study on the treatment of femur head necrosis with tricalcium phosphate and platelet-rich plasma. Zhongguo Xiu Fu Chong Jian Wai Ke Za Zhi.

[11] Latalski M, Elbatrawy YA, Thabet AM (2011). Enhancing bone healing during distraction osteogenesis with platelet-rich plasma. Injury.

[12] Curi MM, Cossolin GS, Koga DH (2011). Bisphosphonate-related osteonecrosis of the jaws - an initial case series report of treatment combining partial bone resection and autologous platelet-rich plasma. J Oral Maxillofac Surg.

[13] Scala M, Gipponi M, Mereu P (2010). Regeneration of mandibular osteoradionecrosis defect with platelet rich plasma gel. In Vivo.

[14] Klein-Nulend J, van der Plas A, Semeins CM (1995). Sensitivity of osteocytes to biomechanical stress in vitro. FASEB J.

[15] Owan I, Burr DB, Turner CH (1997). Mechanotransduction in bone: osteoblasts are more responsive to fluid forces than mechanical strain. Am J Physiol.

[16] Bakker AD, Soejima K, Klein-Nulend J, Burger EH (2001). The production of nitric oxide and prostaglandin E(2) by primary bone cells is shear stress dependent. J Biomech.

[17] Wang Y, Kim UJ, Blasioli DJ (2005). In vitro cartilage tissue engineering with 3D porous aqueous-derived silk scaffolds and mesenchymal stem cells. Biomaterials.

[18] Wang W, Itaka K, Ohba S (2009). 3D spheroid culture system on micropatterned substrates for improved differentiation efficiency of multipotent mesenchymal stem cells. Biomaterials.

[19] Sun S, Ren Q, Wang D, Zhang L, Wu S, Sun XT (2011). Repairing cartilage defects using chondrocyte and osteoblast composites developed using a bioreactor. Chin Med J (Engl).

